# Hierarchically Biporous Wick Vapor Chamber With Micro/Nano Condenser for Exceeding 600 W/cm^2^ Heat Dissipation

**DOI:** 10.1002/smll.202509482

**Published:** 2026-06-11

**Authors:** Ya‐Nan Li, Run‐Sheng Qi, Yawen Shi, Zeng‐Yao Li, Xinpeng Zhao

**Affiliations:** ^1^ Key Laboratory of Thermo‐Fluid Science and Engineering Ministry of Education Xi'an Jiaotong University Xi'an P. R. China; ^2^ Key Laboratory of Environment and Genes Related to Diseases Ministry of Education Xi'an Jiaotong University Xi'an P. R. China; ^3^ Department of Mechanical Engineering Michigan State University East Lansing Michigan USA

**Keywords:** biporous wick, micro/nano‐structured condenser, phase change heat transfer, thermal management, vapor chamber

## Abstract

The rapid advancement of high‐power electronic devices has created an urgent need for more effective thermal management solutions, as inadequate heat dissipation severely limits device performance and reliability. Conventional vapor chambers could dissipate heat efficiently via liquid‐vapor phase change heat transfer but are limited due to capillary pressure‐permeability trade‐off and the long, tortuous condensate return path, resulting in insufficient liquid supply under high thermal loads and making them unsuitable for next‐generation high‐power electronics. Herein, we developed a vapor chamber for high heat flux dissipation by incorporating a hierarchically biporous superhydrophilic evaporator wick for enhanced capillary‐driven liquid supply, along with a superhydrophobic micro/nano‐structured condenser surface that enables dropwise condensation and facilitates condensate return. This synergistic design ensures a continuous and efficient liquid supply, achieving a minimum overall thermal resistance of ∼0.17°C/W, a minimum thermal resistance of temperature uniformity of ∼0.02°C/W, and a critical heat dissipation flux exceeding 600 W/cm^2—^even under gravity‐opposed conditions—showing significant enhancement compared to previous vapor chamber designs. This work provides a promising solution for next‐generation high‐power electronics requiring extreme heat flux dissipation.

## Graphical abstract

An integrated vapor chamber couples a hierarchically biporous copper wick with a micro/nano‐textured superhydrophobic condenser to form a self‐sustained liquid–vapor loop. The device dissipates extreme heat fluxes above 600 W/cm^2^, even when the condenser is above the heater, by overcoming the permeability‐capillarity trade‐off in conventional vapor chambers. This breakthrough enables thermal management for next‐generation high‐power electronics requiring extreme heat flux handling.

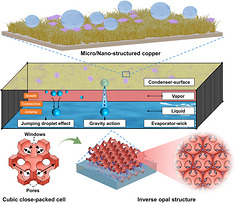

## 1 Introduction

High‐power electronics, such as GaN/SiC power chip systems and integrated power modules, face critical challenges in dissipating extreme heat flux, often reaching up to 500 W/cm^2^ [1, 2, 3]. Insufficient heat dissipation and uneven temperature distribution can severely degrade the device performance and shorten their operational lifespan [4, 5, 6]. Therefore, effective thermal management is essential to ensure system reliability. Vapor chambers, which are passive two‐phase cooling systems, provide a promising solution to efficiently manage heat in high‐power electronic systems by utilizing liquid‐vapor phase‐change heat transfer to dissipate heat from a small heat source to a large heat sink [7, 8, 9]. These systems operate through a continuous cycle: liquid evaporates in the heating area (evaporator), vapor travels to the cooling area (condenser) where it releases latent heat, and the condensed liquid returns to the evaporator through a wick structure and evaporates again. However, at extreme heat loads, a critical limitation arises—when liquid replenishment cannot keep pace with evaporation, the evaporator becomes locally dry, disrupting phase‐change heat transfer and triggering a sharp increase in evaporator temperature. This condition, known as dryout, marks the onset of critical heat flux (CHF)—the maximum heat flux beyond which insufficient liquid return leads to rapid deterioration of thermal dissipation performance. To achieve a high CHF, vapor chamber wicks require improved wicking performance—characterized by permeability and effective capillary radius—to prevent dryout by efficiently returning and distributing liquid across phase change sites within the evaporator. In addition, the liquid return path from condenser to evaporator must be reduced to minimize flow resistance. A fundamental contradiction exists in wick design: achieving high capillary pressure for driving liquid transport needs small pores, while enhanced permeability for minimizing flow resistance demands larger pores, creating a critical trade‐off that limits overall system performance. Therefore, conventional vapor chambers, including single‐sintered powder wicks, mesh wicks, and groove wicks [10, 11, 12], can only achieve a maximum CHF of ∼200 W/cm^2^.

To address the trade‐off between capillary pressure and permeability, various composite structures, including powder‐groove wicks [13, 14, 15, 16, 17], mesh‐groove wicks [18, 19, 20, 21], and powder‐mesh wicks [22, 23] have been demonstrated to enhance capillary pressure while maintaining a high permeability. However, the reported composite structures, with a maximum CHF under 400 W/cm^2^, fail to meet increasing thermal management demands and are difficult to manufacture and scale due to their complex fabrication. Recent advances in surface modification technologies (e.g., etching [24], oxidation [17], deposition [25], laser processing [26], and plasma processing [27]) have introduced new possibilities for enhancing the wicking performance of various nanostructures [28, 29, 30, 31]. For example, Damoulakis et al. [32] developed a vapor chamber utilizing precise surface‐wettability patterning to generate wetting forces and transport liquid from condenser to evaporator efficiently, achieving a critical heat flux of 220 W/cm^2^, and a minimum thermal resistance of 0.18°C/W. Lv et al. [24] reported a vapor chamber utilizing a superhydrophilic copper mesh wick, treated through a combined etching and sintering process to optimize both capillary pressure and permeability, achieving a maximum heat flux of 490 W/cm^2^. Despite these advancements, the intrinsic trade‐off between capillary pressure and permeability still prevents the existing vapor chambers from effectively managing heat fluxes > 500 W/cm^2^ required by next‐generation electronic devices.

**FIGURE 1:**
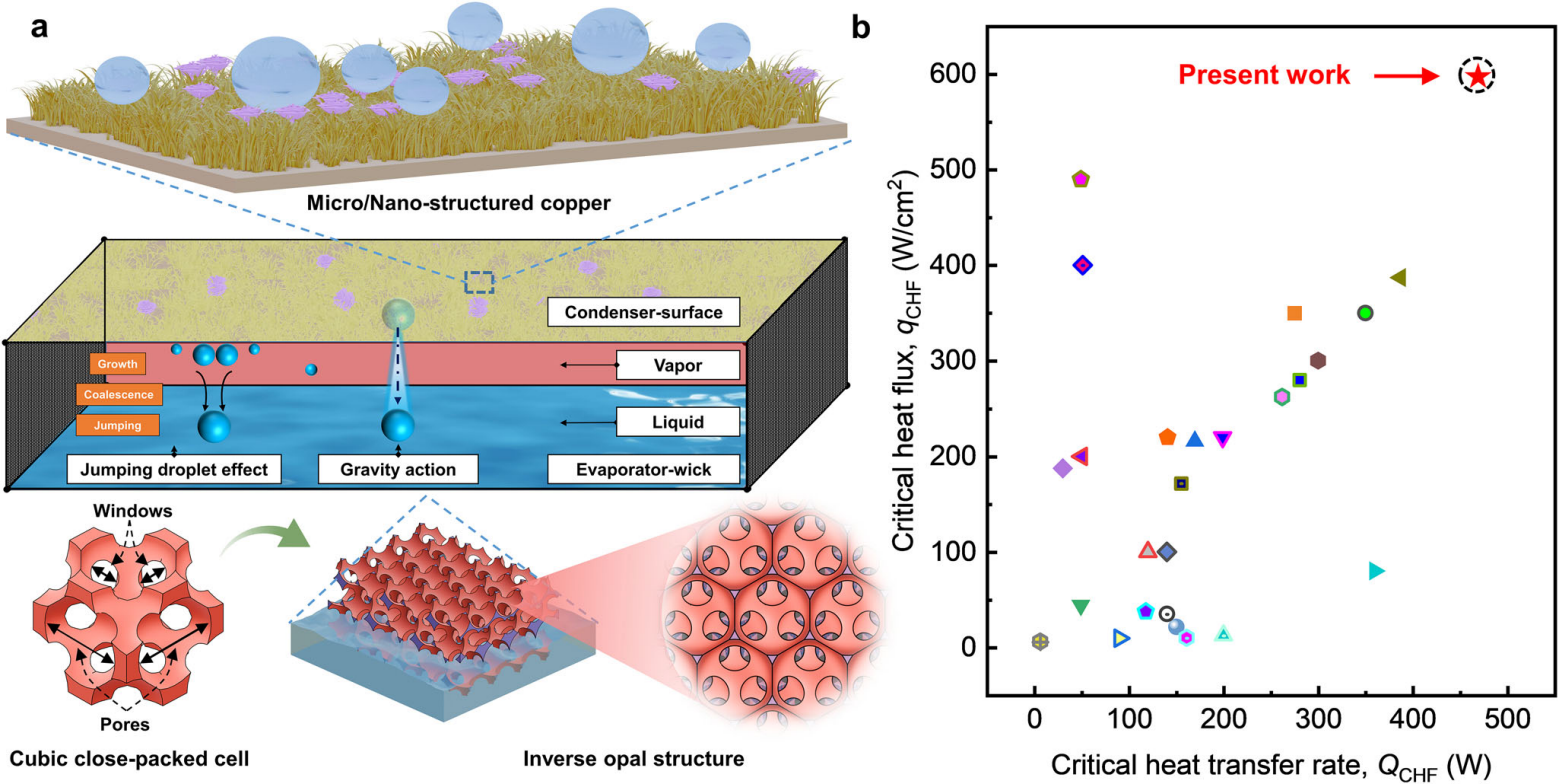
Geometric design and heat dissipation performance of the vapor chamber. (a) Schematic of the vapor chamber, featuring a hierarchically biporous superhydrophilic wick in the evaporator (bottom) and a superhydrophobic micro/nano‐structured copper surface in the condenser (top). Liquid water at the bottom absorbs heat and vaporizes. The vapor then rises to the top, where it condenses, releasing heat into the environment. The condensed liquid then returns to the bottom evaporator through gravity and the “jumping droplet” effect. (b) Comparison of the critical heat fluxes (CHF) and heat transfer rates of the vapor chambers with various wick configurations [2, 14, 24, 25, 32, 33, 34, 35, 36, 37, 38, 39, 40, 41, 42, 43, 44, 45, 46, 47, 48, 49, 50, 51, 52]. Our design achieves a CHF of > 600 W/cm^2^, which is higher than the previously reported values for vapor chambers, while maintaining orientation insensitivity.

In this work, we designed and demonstrated a high‐performance vapor chamber that overcomes traditional wicking limitations by incorporating a hierarchically biporous, superhydrophilic evaporator wick that provides high capillary pressure and permeability for efficient liquid transport, combined with a superhydrophobic micro/nano‐structured condenser to facilitate liquid return via “jumping droplet” effect (Figure 1). The evaporator wick, fabricated using inverse copper opal, features a biporous architecture: (1) large cubic close‐packed pores (∼10 µm) offer high permeability for fluid transport from the cooling area to heating area, and (2) small pores (∼1.6 µm) between large pores generate high capillary pressure for sustained liquid supply, effectively resolving the conventional trade‐off between permeability and capillary pressure. The micro/nano‐structured copper condenser (Figure 1a), with its hierarchically rough surface of microscale flower‐like and nanoscale grass‐like features, promotes dropwise condensation and spontaneous liquid return, creating a synergistic effect that dramatically enhances the continuous phase‐change cycle. Our experimental results demonstrate exceptional heat dissipation capabilities, with the vapor chamber achieving a critical heat flux exceeding 600 W/cm^2^ at all inclinations, showing significant enhancement compared to other vapor chambers (Figure 1b). Notably, even under gravity‐opposed conditions, the vapor chamber maintains robust thermal performance, achieving a minimum overall thermal resistance of 0.17°C/W and superior temperature uniformity with a thermal resistance of 0.02°C/W. Furthermore, the vapor chamber and its key components, including the superhydrophilic evaporator wick and superhydrophobic condenser surface, show stable performance during the 72‐h continuous operation tests. The demonstrated performance improvements and fundamental insights into biporous wicking structures provide a promising pathway for advancing thermal management technologies in increasingly power‐dense electronic systems.

## 2 Results and Discussion

### 2.1 Structure Fabrication and Characterization

**FIGURE 2:**
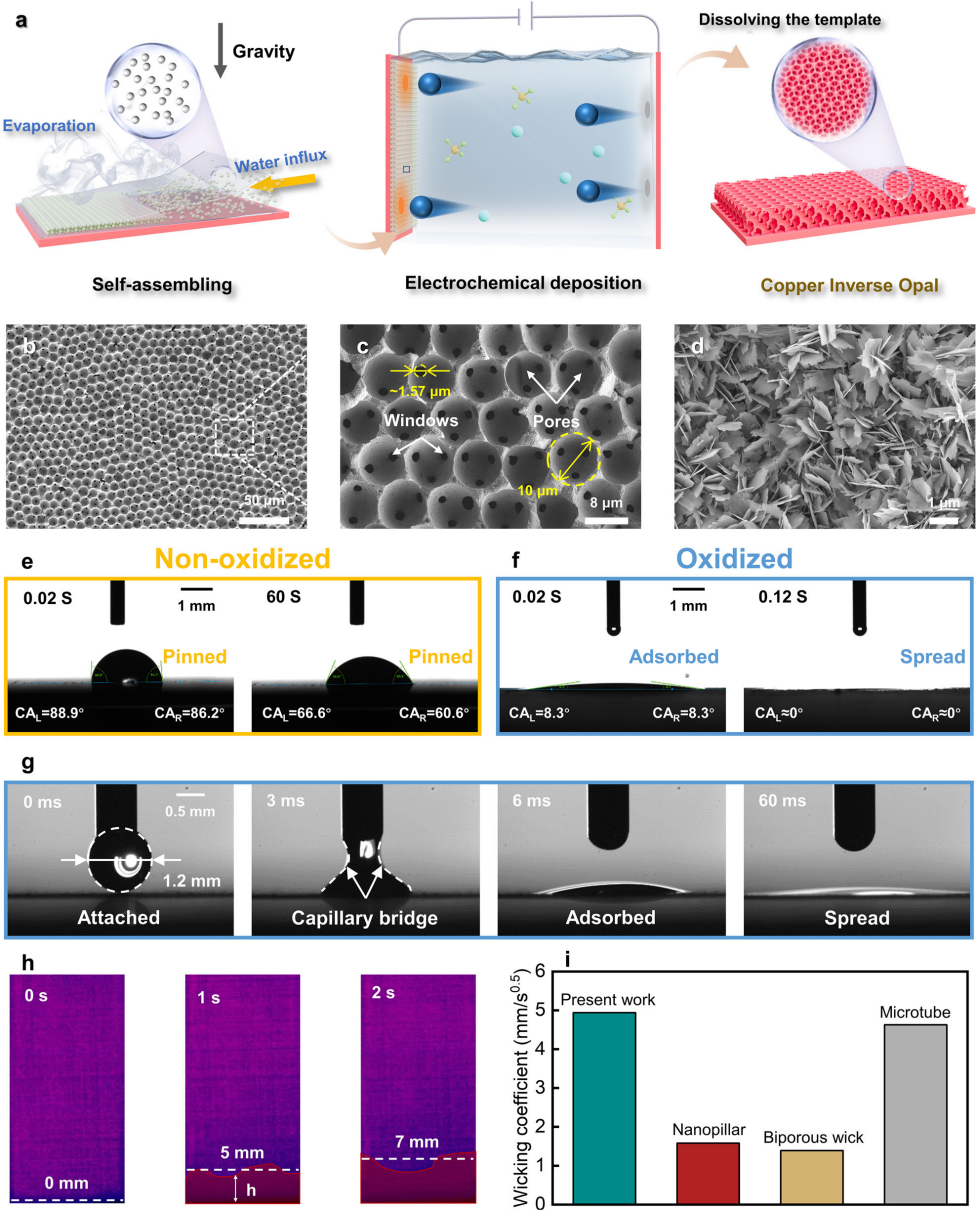
Fabrication and characterization of the copper evaporator wick. (a) Schematic of the fabrication process of the copper inverse opal structure. The structure is created by the electrochemical deposition of copper into a sintered polystyrene opal template, followed by a chemical oxidation treatment to achieve superhydrophilic properties. SEM images of the (b, c) non‐oxidized copper inverse opal, and (d) the oxidized superhydrophilic copper inverse opal surface. The regular copper inverse opal exhibits a biporous structure, characterized by large pores with a diameter of ∼10 µm and small interconnecting pores with a diameter of ∼1.6 µm. Water contact angle tests of (e) non‐oxidized, and (f) oxidized copper inverse opal. (g) Time‐lapse images showing the liquid suction process on the oxidized superhydrophilic copper inverse opal at 0° orientation. (h) Visualization of capillary rise height of the copper inverse opal using infrared imaging to track liquid transport, the red zone indicates the wetted region. (i) Comparison of the wicking coefficient between our wick and conventional designs, including nanopillar wick [53], biporous wick [3], and microtube wick [54].

To simultaneously achieve high permeability and high capillary pressure, we engineered a three‐dimensional ordered biporous microstructure using an inverse opal structure. This structure features large pores (∼10 µm) arranged in a cubic close‐packed configuration for efficient fluid transport, interconnected by small pores (∼1.6 µm) that generate strong capillary pressure [33, 34, 35]. In a typical process, copper was electrochemically deposited into a sintered polystyrene (PS) opal template to fill the voids, followed by template removal to yield the copper inverse opal (Figure 2a). The large pore diameter (∼10 µm) was precisely controlled by the PS sphere size, while the small pore diameter was ∼1.6 µm by sintering the template at 105°C for 90 min, resulting in a porosity of ∼0.77 (Figure 2b,c, Figure S1b). To further enhance the wick's superhydrophilicity, we applied a chemical oxidation treatment, which transformed the copper surface into a copper oxide layer with higher surface energy (Figure S1c,d) and generated a hierarchical nanotexture to enhance the roughness (Figure 2d).

We compared the wettability transition induced by oxidation in Figure 2e,f by performing contact angle measurements using a drop‐shape analyzer (KRUSS DSA100S). As shown, a water droplet (∼3.9 µL) remains pinned on the non‐oxidized surface with a contact angle of approximately 60–66°, while on the oxidized surface, it is adsorbed quickly and then spreads into a liquid film (contact angle approaching 0°), confirming robust superhydrophilic property. To further evaluate the wicking performance of the oxidized surface, we analyzed the suction processes of water droplets on the surface at 0°, 90°, and 180° inclinations through a goniometer equipped with a high‐speed camera (Figure S2a, Figure 2g, Video S1). Upon contact with the surface, the droplet is rapidly drawn into the structural voids, forming a temporary liquid capillary bridge between the superhydrophilic copper inverse opal and the stainless needle. The liquid capillary bridge breaks within 6 ms, followed by spontaneous droplet spreading into a thin film across an extended area within 60 ms. Notably, this rapid wicking behavior occurs consistently at all tested inclinations. The complete thin‐film spreading occurs within ∼60 ms, which is 3 to 10 times faster than conventional surfaces [36, 37, 38] that typically require hundreds of milliseconds, demonstrating significantly improved capillarity and permeability (Figure 2g, Figure S2b,c). To quantitatively evaluate the combined permeability and capillarity of the copper inverse opal, we conducted a capillary rise test and analyzed the height‐time relationship using the Lucas‐Washburn equation to determine the effective wicking coefficient. Figure 2h shows the infrared imaging of the capillary rise test, where the capillary pressure drives the liquid spontaneously upward along the wick, reaching a height of ∼7 mm within 2 s. Comparison of wicking performance (Figure 2i) shows our design achieves a higher effective wicking coefficient of 4.9 mm/s^0.5^, outperforming conventional wick structures [3, 53, 54].

**FIGURE 3:**
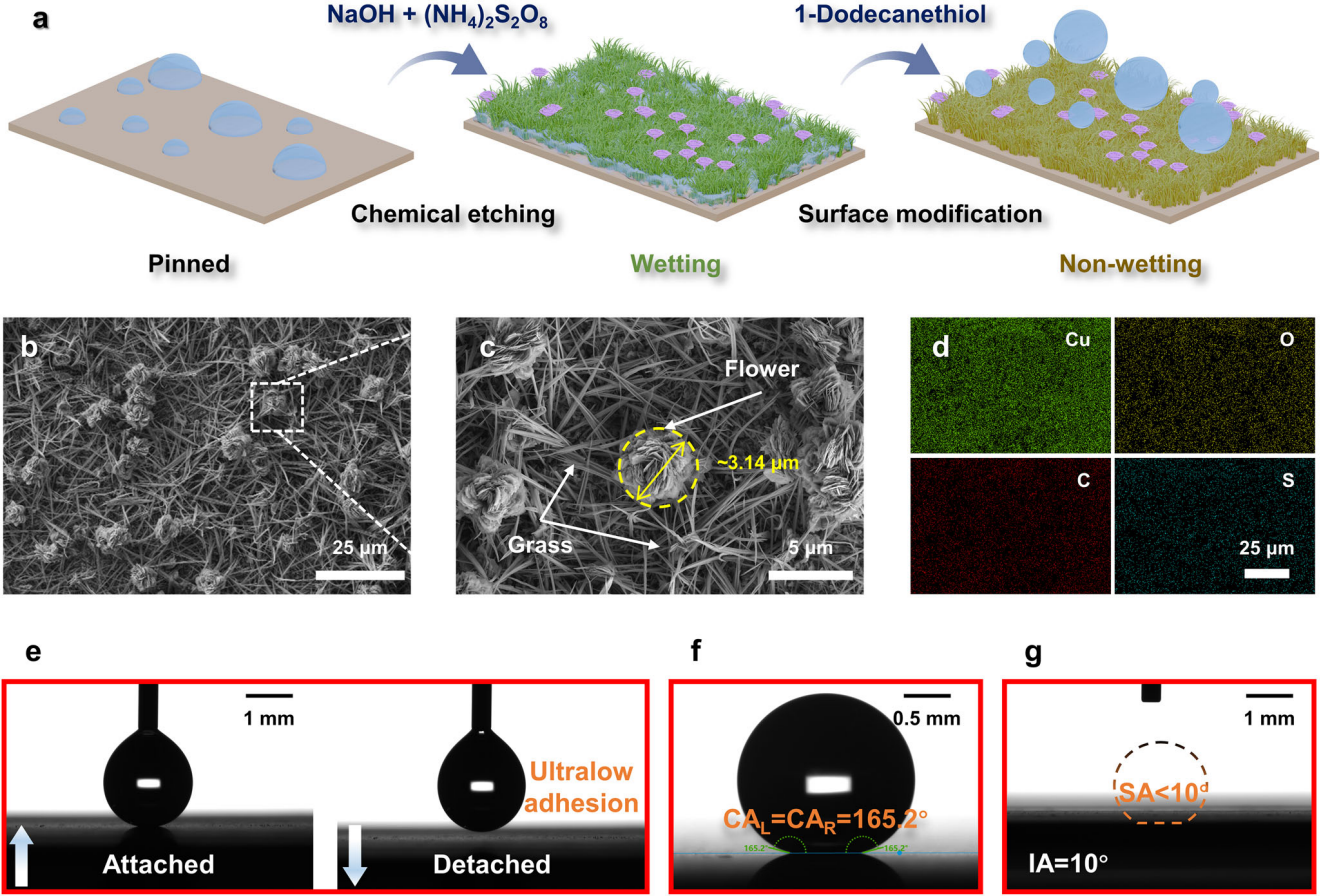
Fabrication and characterization of micro/nano‐structured copper surface of the condenser. (a) Schematic illustration of the fabrication process: chemical etching creates interwoven nanostructures to enhance surface roughness, followed by low‐surface‐energy 1‐dodecanethiol modification to achieve superhydrophobicity. (b, c) SEM images of the superhydrophobic surface. (d) Elemental mapping of the condenser surface. (e) Adhesion test, (f) water contact angle test, and (g) sliding angle measurement of the micro/nano‐structured copper surface.

We employed a micro/nano‐structured superhydrophobic copper as the condenser surface to enable superior dropwise condensation, which has been reported to achieve a heat transfer coefficient ten times higher than that of filmwise condensation on conventional surfaces [5, 30]. Additionally, the superhydrophobic condenser surface has been reported to enable the “jumping droplet” effect, where condensate droplets spontaneously self‐propel away from the condenser along the vertical direction [5, 42, 55]. This creates a more efficient return path to the evaporator, as the vertical trajectory represents the shortest distance between the parallel plates of the condenser and evaporator. The superhydrophobic surface was fabricated through a two‐step process: (1) chemical etching to create interwoven microscale flower‐like and nanoscale grass‐like structures for enhanced roughness (Figure 3a‐c), followed by (2) surface modification with low‐surface‐energy 1‐dodecanethiol to establish superhydrophobicity (Figure 3d), synergistically enabling Cassie‐Baxter wetting through air entrapment at the liquid‐solid interface.

We confirmed the superhydrophobic characteristics of the micro/nano‐structured copper surface through systematic wettability measurements, including water droplet adhesion tests, contact angle (CA), and sliding angle (SA) characterization (Figure 3e, Figure S3). The adhesion test using water droplets (∼4.6 µL) demonstrated the ultra‐low adhesion, with droplets readily detaching from the surface (Video S2). As shown in Figure 3e‐g, contact angle measurements revealed a high contact angle (∼165.2°) and low sliding angle (less than 10°), characteristic of a Cassie state (also known as the “lotus” state) [56, 57, 58]. These characteristics, consistent with the Cassie‐Baxter model of composite liquid/air and liquid/solid interfaces, confirm the superhydrophobic nature of the fabricated surface.

### 2.2 Thermal Dissipation Performance

**FIGURE 4:**
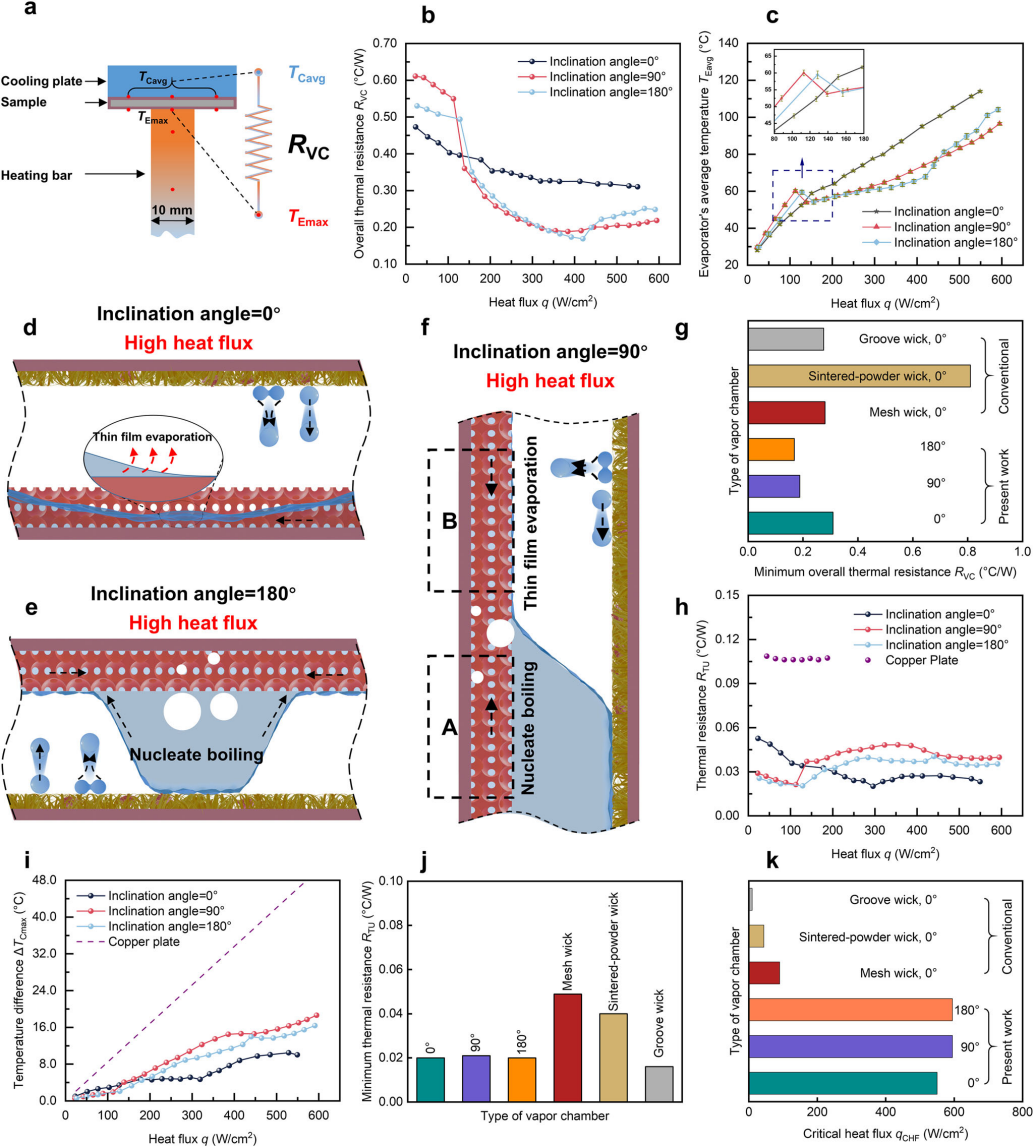
Thermal dissipation performance of the vapor chamber. (a) Schematic of the vapor chamber thermal dissipation performance testing system at 0°. (b) Overall thermal resistance *R*_VC_ between the evaporator and condenser as a function of heat flux and inclination, which is calculated using the maximum temperature of evaporator *T*_Emax_ and the average temperature of condenser *T*_Cavg_. (c) Average temperature of the evaporator *T*_Eavg_ as a function of heat flux and inclination, which is used to characterize the thermal response associated with the evaporation‐to‐boiling transition. The decrease in *T*_Eavg_ by ∼8°C near ∼120 W/cm^2^ at 90° and 180° inclinations, despite increasing heat flux, supports the transition from evaporation‐dominated phase change heat transfer to nucleate boiling. Phase distribution and liquid‐vapor phase change behavior at high heat flux (e.g., ∼400 W/cm^2^) at (d) 0°, (e) 180°, and (f) 90°, respectively. At this condition, boiling heat transfer dominates the phase‐change phenomenon at 180° and 90° inclinations. (g) Comparison of the minimum overall thermal resistance *R*_VC_ between our vapor chamber and conventional designs. (h) Temperature uniformity of the condenser *R*_TU_ as a function of heat flux and inclination. (i) Maximum condenser temperature difference Δ*T*_Cmax_ as a function of heat flux and inclination. (j) Comparison of the temperature uniformity of the condenser *R*_TU_ among our vapor chamber and conventional designs [24, 35, 44]. (k) Critical heat flux between our vapor chamber (> 600 W/cm^2^) and conventional designs.

We integrated the hierarchically biporous wick (80 mm × 45 mm× ∼0.1 mm) evaporator (Figure 2) with the superhydrophobic micro/nano‐structured copper condenser of matching dimensions (Figure 3) to build a vapor chamber (Figure S4a). A home‐made system, including heating/cooling elements, thermal insulation, and data acquisition (Figures S5 and S6), was used to evaluate the thermal dissipation performance of the vapor chamber. As Figure 4a shows, during testing, a joule‐heated cylindrical bar with a diameter of 10 mm (∼78.5 mm^2^ heating area) was used on the evaporator side to mimic the local high heat flux of high‐power electronic devices, which is a widely adopted method for its well‐defined heating area, precise power control, and excellent repeatability [21, 22]. A water‐cooled flat plate on the condenser side was used to remove the heat transferred from the evaporator, with the inlet cooling water maintained at 20°C and a flow rate of 13.4 L/min which are typical values adopted in the prior tests. The thermal dissipation performance of the vapor chamber was evaluated based on (1) the overall thermal resistance between the evaporator and condenser (*R*_VC_), (2) the temperature uniformity of the condenser, and (3) the critical heat flux (CHF) from the evaporator to the condenser [4, 9, 10, 11]. The overall thermal resistance, *R*_VC_, is calculated using the common method, which is given by:

(1) $$\begin{array}{c} \;R_{\mathrm{VC}}=\frac{T_{\mathrm{Emax}}-T_{\mathrm{Cavg}}}{Q} \end{array}$$

where *T*_Emax_ is the maximum temperature of the evaporator and is measured by the thermocouple located at the evaporator center. *T*_Cavg_ is the average temperature of the condenser, obtained from the distributed thermocouples. *Q* is the heat transfer rate from the heating area to the cooling area. The temperature uniformity of the condenser is characterized by the maximum temperature difference across the condenser, Δ*T*_Cmax_, and the corresponding thermal resistance (*R*_TU_), defined as

(2) $$\begin{array}{c} \Delta \;T_{\mathrm{Cmax}}=T_{\mathrm{Cmax}}\;-T_{\mathrm{Cmin}} \end{array}$$

(3) $$\begin{array}{c} \;R_{\mathrm{TU}}=\frac{\Delta T_{\mathrm{Cmax}}}{Q} \end{array}$$

where *T*_Cmax_ and *T*_Cmin_ are the maximum and minimum temperatures of the condenser. The CHF represents the maximum heat flux at which insufficient liquid return leads to evaporator dryout, causing a rapid increase in the overall thermal resistance *R*_VC_ and the evaporator temperature.

Figure 4b,c shows the overall thermal resistance (*R*_VC_) of the built vapor chamber and evaporator average temperature as a function of heat flux (∼20–∼600 W/cm^2^) at three typical inclinations of 0°, 180°, and 90°. As Figure 4b shows, the overall thermal resistance *R*_VC_ decreases with increasing heat flux *q* in all cases, consistent with the typical thermal‐resistance trend of vapor chambers and other two‐phase heat transfer systems [59, 60], while its detailed evolution still varies with inclination. At 0° inclination (i.e., horizontal), performance varies across three distinct regimes. When heat flux (i.e., < 100 W/cm^2^) is low, gravity creates a thick liquid film (Figure S7a), increasing the conductive thermal resistance from the heating area to the planar liquid‐vapor interface where evaporation occurs, resulting in a high *R*_VC_ ∼0.47°C/W at ∼23 W/cm^2^. As the heat flux increases, the liquid film gradually thins with more liquid being heated and evaporated, reducing *R*_VC_ from 0.47°C/W to 0.39°C/W (∼23–∼180 W/cm^2^). Notably, at ∼200 W/cm^2^, the liquid film recedes into the wick (Figure 4d), enhancing thin film evaporation through meniscus formation, which increases the evaporation area and further reduces *R*_VC_ from 0.39°C/W at ∼180 W/cm^2^ to 0.35°C/W. From ∼200 to ∼550 W/cm^2^, *R*_VC_ continues to linearly decrease to ∼0.31°C/W because liquid is sufficiently supplied from the condenser to evaporator via gravity and “jumping droplet” effect, and then reaches the heating area driven by capillary pressure generated in the superhydrophilic wick. The behavior at 180° (inverted) and 90° (vertical) inclinations follows a different pattern. Initially, liquid pools form on the superhydrophobic condenser (180°, Figure S7b) or at the chamber bottom due to gravity (90°, Figure S7c), creating a 1‐mm‐thick capillary bridge. At low heat flux (e.g., < 100 W/cm^2^), heat transfer from the evaporator to the condenser is dominated by conduction through liquid pools, resulting in a large *R*_VC_ at ∼20 W/cm^2^ (0.53°C/W at 180°, and 0.61°C/W at 90°). With increasing heat flux up to ∼120 W/cm^2^, enhanced thin film evaporation in regions without liquid pools improves the overall heat dissipation performance and reduces *R*_VC_ to 0.50°C/W (180°) and 0.54°C/W (90°). As the heat flux continues to rise, the evaporator temperature increases, eventually reaching the onset of nucleate boiling in the vapor chamber. Beyond ∼120 W/cm^2^, nucleate boiling initiates in the porous wick, significantly improving heat dissipation, dramatically reducing *R*_VC_ to 0.34°C/W at 180° (∼420 W/cm^2^), and 0.35°C/W at 90° (∼390 W/cm^2^), as shown in Figure 4e,f. The decrease in the average evaporator temperature *T*_Eavg_ by ∼8°C near ∼120 W/cm^2^ at 90° and 180° inclinations, despite increasing heat input (Figure 4c), is consistent with the reported thermal response associated with the transition from evaporation to nucleate boiling [2]. Besides, this transition is further supported by a noticeable shift in acoustic signals from quiet evaporation to vigorous crackling sounds, marking the onset of nucleate boiling within the chamber (Figure S8). As heat flux further increases, more nucleation sites are activated, enhancing boiling heat transfer coefficient and reducing the overall thermal resistance *R*_VC_ gradually to ∼0.17°C/W at ∼420 W/cm^2^. However, at the heat flux >420 W/cm^2^, excessive liquid vaporization reduces liquid pools and limits the boiling heat transfer, increasing *R*_VC_ to ∼0.25°C/W at 180° and ∼0.23°C/W at 90° when the heat flux is ∼600 W/cm^2^. We note that our vapor chamber sustains nucleate boiling up to ∼600 W/cm^2^ without a boiling crisis, as the efficient wicking performance of the superhydrophilic evaporator wick ensures continuous liquid supply, preventing dryout. Figure 4g compares the minimum *R*_VC_ of our vapor chamber at 0°, 90°, and 180° positions with the state‐of‐the‐art designs, showing its superior *R*_VC_ (e.g., ∼0.17°C/W at 180°, ∼420 W/cm^2^) across different operating conditions over conventional vapor chambers with groove wick (∼0.27°C/W) [44], sintered‐powder wick (∼0.81°C/W) [35], and mesh wick (∼0.28°C/W) [61].

Figure 4h,i shows the temperature uniformity across the condenser characterized by the thermal resistance *R*_TU_ and the corresponding maximum temperature difference Δ*T*_Cmax_. Similar to Figure 4b,c, the temperature uniformity trends with heat flux vary based on the inclination angle of the vapor chamber. At 0° (horizontal) orientation, the temperature uniformity shows a straightforward improvement with increasing heat flux. The initial high *R*_TU_ (∼0.05°C/W at ∼23 W/cm^2^) decreases steadily to ∼0.02°C/W at ∼550 W/cm^2^, primarily due to the widening of vapor channels from the evaporator to the condenser as the liquid film thins. The vapor flow becomes less restricted, resulting in reduced pressure drop and more uniform temperature distribution across the condenser. For 180° (inverted) and 90° (vertical) positions, the temperature uniformity exhibits a two‐stage thermal response associated with the transition from evaporation to nucleate boiling. Below the boiling onset (∼120 W/cm^2^), *R*_TU_ maintains almost a constant value of ∼0.03°C/W, supported by gravity‐driven liquid pools. However, the initiation of nucleate boiling disrupts this stability, causing *R*_TU_ to briefly increase due to heightened vapor pressure drop from bubble nucleation, growth, departure, and collapse at the liquid‐vapor interface. As a result, *R*_TU_ increases from ∼0.02°C/W at 113 W/cm^2^ to ∼0.04°C/W at 139 W/cm^2^ (at 90°) and from ∼0.02°C/W at 128 W/cm^2^ to ∼0.03°C/W at 156 W/cm^2^ (at 180°). For comparison, we also measured the temperature uniformity of a copper plate of the same dimensions as the vapor chamber. As shown in Figure 4h, the vapor chamber achieves a more uniform temperature distribution, with the *R*_TU_ of the vapor chamber ranging from ∼0.05°C/W to ∼0.02°C/W, compared to ∼0.1°C/W for the copper plate. At 550 W/cm^2^, the temperature difference of the vapor chamber (8–15°C) is 30–37°C lower than that of the copper plate (> 45°C, Figure 4i). Furthermore, our chamber shows competitive temperature uniformity compared to conventional designs (Figure 4j), achieving a minimum *R*_TU_ of 0.02°C/W, similar to groove wick (∼0.016°C/W) [44], sintered‐powder wick (∼0.041°C/W) [35], and mesh wick (∼0.049°C/W) designs [24].

Effective cooling of high‐power electronic devices demands a vapor chamber that maintains low overall thermal resistance and uniform temperature distribution at high heat flux. Our designed vapor chambers demonstrate exceptional performance in this regard, achieving a maximum heat flux of >550 W/cm^2^ at 0°, and >600 W/cm^2^ at 90° and 180° orientations(Figure 4k)—substantially outperforming conventional designs including groove wick (∼9.35 W/cm^2^) [44], sintered‐powder wick (∼44 W/cm^2^) [35], mesh wick (∼90 W/cm^2^) [61], and nano‐engineered wicks (Figure 4k, Figure 1b, Table S2). At the maximum heat flux tested, no dryout signature was observed, as indicated by stable evaporator temperature and thermal dissipation performance. The test was terminated due to safety constraints from the heating block's surrounding PTFE insulation (∼400°C), making the reported CHF a conservative estimate. With a critical heat flux of > 600 W/cm^2^ and a heat transfer rate of > 470 W, our vapor chamber exhibits significantly enhanced heat dissipation capacity compared to previous vapor chamber designs. This exceptional performance is attributed to two key features: the efficient wicking performance of the superhydrophilic evaporator wick (Figure 2) and the enhanced liquid return from the superhydrophobic condenser via the “jumping droplet” effect (Figure 3).

To provide a preliminary durability assessment of the vapor chamber, we evaluated the operational stability of the three key components: the superhydrophilic evaporator wick, the superhydrophobic condenser surface, and the assembled vapor chamber device. Specifically, a pool boiling setup was employed to assess the operation stability of the superhydrophilic wick. The results show that the wick maintained stable boiling heat transfer performance under a heat flux of ∼175 W/cm^2^ for over 72 h (Figure S9a‐c). The durability of the superhydrophobic surface was also confirmed, as it retained a water contact angle of >150° after being immersed in water for more than 72 h (Figure S9d‐f). Furthermore, the vapor chamber can maintain a stable overall thermal resistance of ∼0.22°C/W under ∼570 W/cm^2^ heating condition (180° inclination) for over 72 h (Figure S9g‐i). Although these results demonstrate stable phase‐change heat transfer performance during the 72‐h continuous tests, further extended lifetime evaluations over longer durations, together with thermal cycling, mechanical reliability assessment, and device‐level integration tests, are still required to advance the vapor chamber from laboratory‐scale demonstration toward real‐world industrial applications.

## 3 Conclusions

In summary, we developed a high‐performance vapor chamber by integrating a hierarchically biporous superhydrophilic evaporator wick (∼10 µm for big pores, ∼1.6 µm for small pores) and a superhydrophobic micro/nano‐structured copper condenser (∼165° contact angle, <10° sliding angle). The superhydrophilic biporous evaporator wick enables rapid liquid spreading (∼60 ms), enhancing wicking performance (4.9 mm/s^0.5^), and efficient fluid transport to the heating area. Meanwhile, superhydrophobic micro/nano‐structured copper condenser promotes dropwise condensation and facilitates condensate return through the “jumping droplet” effect. The vapor chamber demonstrates superior heat dissipation capacity (i.e., > 600 W/cm^2^) and maintains stable operation across various inclinations, including gravity‐opposed conditions (e.g., evaporator below the condenser). Additionally, the vapor chamber exhibits a relatively low overall thermal resistance (∼0.17°C/W) and a high temperature uniformity (∼0.02°C/W), helping to mitigate thermal stress and overheating, thereby potentially extending the service life of electronic devices. The vapor chamber exhibits superior thermal dissipation performance and stable operation during the 72‐h continuous tests, indicating its potential for cooling high‐power electronic devices, including GaN/SiC power chip systems, central processing units (CPUs), graphics processing units (GPUs), and integrated power modules. Additionally, its applicability extends to harsh environments, such as radar transmitters, aerospace systems, and other high‐performance electronics, where efficient thermal management is crucial.

## 4 Methods

### 4.1 Materials and Chemicals

Commercially available T2 oxygen‐free copper plates (> 99.9%) were used as substrates for the fabrication of the evaporator and condenser. Polystyrene (PS) microspheres (Suzhou Knowledge & Benefit Sphere Tech. Co., Ltd.) were used for self‐assembly of opal templates. NaOH (> 98%, Macklin) and (NH4)_2_S_2_O_8_ (> 98.5%, Macklin) were used for chemical oxidation and etching. H_2_SO_4_ (> 98%, Sinopharm) and CuSO_4_ (> 98%, Aladdin) were used for electrochemical deposition. 1‐Dodecanethiol (∼98%, Macklin) and ethanol (∼99.5%, Macklin) were used for superhydrophobic surface modification.

### 4.2 Fabrication of the Evaporator Wick

The inverse opal evaporator wick was fabricated by depositing a copper inverse opal structure onto a copper substrate, followed by chemical oxidation. A 99.99% pure copper substrate (45 mm × 80 mm, Shenzhen Beisi Technology Co., Ltd.) was first polished with sandpapers of grits 400–5000, then cleaned with acetone, 5% diluted sulfuric acid, ethanol, and DI water, and dried under nitrogen (N_2_). Next, the cleaned substrate was then subjected to oxygen plasma treatment to enhance the adhesion between the substrate and the polystyrene (PS) microspheres during self‐assembly. Afterward, a 1:9 diluted PS/water suspension (10% w/v, Suzhou Knowledge & Benefit Sphere Tech. Co., Ltd.) was evenly spread on the substrate. The substrate was then heated to 45 ± 0.5°C to expedite evaporation, allowing PS microspheres to self‐assemble into a cubic close‐packed template, followed by sintering the template at ∼105°C for 90 min. Copper was subsequently electrodeposited into the template at 20 mA/cm^2^ for 2 h using 0.6 M CuSO_4_ and 30 mM H_2_SO_4_, forming a ∼100 µm thick copper inverse opal layer. Finally, the PS template was dissolved in tetrahydrofuran for 48 h. The copper structure was etched in 2.5 M NaOH and 0.13 M (NH_4_)_2_S_2_O_8_ for 30 min to create rough Cu(OH)_2_ on the surface, then dried in a vacuum to achieve the evaporator wick.

### 4.3 Fabrication of the Condenser Surface

The superhydrophobic micro/nano‐structured condenser surface was fabricated via in‐situ CuO growth and surface modification. First, a copper substrate, identical to the evaporator wick, was polished, cleaned, and then etched in 2.5 M NaOH and 0.13 M (NH_4_)_2_S_2_O_8_ for 8 min at 35°C, producing Cu(OH)_2_ nanostructures. Next, the substrate was oxidized at 160 ± 5°C for 6 h to convert Cu(OH)_2_ to CuO, and strengthen the structure. Finally, it was then immersed in a 1% v/v 1‐dodecanethiol/ethanol solution for 15 min to reduce surface energy, followed by vacuum drying at 60°C.

### 4.4 Fabrication of the Vapor Chamber

The vapor chamber was constructed from a copper evaporator, a condenser, and a positioning frame (Figure S4a). The evaporator and condenser plates (96 mm × 61 mm ×3 mm) were selected to ensure structural stability without internal supports and to allow reliable machining of the filling port. Together with a 1‐mm‐thick frame, they were sealed together to form a chamber using glue (3M Scotch‐Weld Epoxy Adhesive DP420NS and Agilent Torr Seal). Next, a custom evacuation‐filling system was set up to ensure gas tightness and control the pressure and filling ratio of the working fluid (DI water). The system comprised a vacuum pump for evacuating the chamber, an injector for filling the liquid, a vacuum gauge for monitoring pressure, and a high‐pressure nitrogen cylinder to provide positive pressure. Then, the filling ratio of DI water was controlled by first reducing the system pressure to ∼50 Pa using a vacuum pump (Edwards RV8) while monitoring the vacuum gauge. Degassed DI water, boiled for 30 min and cooled in a vacuum box, was injected into the chamber to achieve a typical filling ratio of ∼50%, defined as the volume of liquid relative to the total chamber volume. After the initial filling, the chamber was evacuated for an additional 10 min to remove any non‐condensable gases. To minimize water loss during this step, the chamber was positioned vertically with the filling port at the top (Figure S4c), and ∼0.3 g of water was intentionally overfilled to compensate for the small, consistent loss observed during evacuation, ensuring the desired filling ratio. Once evacuated, the chamber was sealed and prepared for testing.

### 4.5 Measurements and Characterizations

A SEM (GEMINISEM 500, ZEISS) was used to characterize the morphology and structure of the hierarchically biporous wick and the micro/nano‐structured surface. A contact angle analyzer (DSA100S, KRUSS) was used to measure the water contact angle on the evaporator wick and the condenser surface. A home‐made system with a high‐speed camera (Revealer M230) was used to record the suction processes of water droplets on the evaporator wick (Figure S2a).

### 4.6 Thermal Dissipation Performance Test

A custom‐designed system was used to evaluate the thermal dissipation performance of the vapor chamber (Figure S5). Four joule heating rods, controlled by a programmable power supply (IT6833A, Itech Electronic Co., Ltd.), were adopted as the heat source. The heating bar (10 mm‐diameter column) had two embedded K‐type thermocouples (Omega GG‐K‐30) for temperature measurement. Heat flux and transfer rate were calculated using Fourier's law. The vapor chamber was cooled by a water‐cooling system with a chiller (Thermo Scientific ThermoFlex TF2500) and a finned aluminum cooling block. The water flow rate was ∼13.4 L/min, and the inlet water temperature was ∼20°C (room temperature). A thermal insulation system, using polytetrafluoroethylene (PTFE) blocks and aluminum silicate, minimized heat loss from the heating module and the vapor chamber, and provided structural support. Temperature data was recorded using a Keithley DAQ 6510 with 20 calibrated K‐type thermocouples. A multi‐functional indexing instrument was utilized to achieve the control of inclinations. During the tests, power was increased in 20 W increments, starting from 20 W, until the temperature reached the material's temperature limit (∼330°C for PTFE in this work). At each step, thermal stability was considered achieved when all measured temperatures changed by less than 0.5°C over a 10‐minute period. Data from the last minute was recorded. Detailed data reduction and uncertainty analysis are provided in Equations S1–S16.

## Author Contributions

Y.L. conceived the idea. Z.L. supervised the work. Y.L., R.Q., and Y.S. conceptualized the study. Y.L. and R.Q. designed the solution. Y.L. and Y.S. fabricated the samples. Y.L. and R.Q. carried out the characterization. Y.L. prepared the original draft. Y.L., Z.L., and X.Z. reviewed and edited the manuscript.

## Conflicts of Interest

The authors declare no conflicts of interest.

## Supporting information




**Supporting File 1**: smll74145‐sup‐0001‐SuppMat.pdf.

**Supporting File 2**: smll74145‐sup‐0002‐VideoS1.mp4.

**Supporting File 3**: smll74145‐sup‐0003‐VideoS2.mp4.

## Data Availability

The data that support the findings of this study are available from the corresponding author upon reasonable request.
