# Supplementary material for: Hierarchically Biporous Wick Vapor Chamber With Micro/Nano Condenser for Exceeding 600 W/cm2 Heat Dissipation
Source: Small. 2026 Jun 11;22(44):e09482. doi: 10.1002/smll.202509482 (PMC13450345; doi:10.1002/smll.202509482)
Supplement: Supplementary file 1 [file SMLL-22-e09482-s002.pdf]

Hierarchically Biporous Wick Vapor Chamber with Micro/Nano Condenser for Exceeding 600 W/cm^2 Heat Dissipation

Yanan Li[1], Runsheng Qi[1], Yawen Shi[2], Zengyao Li[1*], Xinpeng Zhao[3*]

1. Key Laboratory of Thermo-Fluid Science and Engineering, Ministry of Education, Xi'an Jiaotong University, Xi'an 710049, P.R. China
2. Key Laboratory of Environment and Genes Related to Diseases, Ministry of Education, Xi'an Jiaotong University, Xi'an 710049, P.R. China
3. Department of Mechanical Engineering, Michigan State University, East Lansing, MI 48824, USA

[*] Email: lizengy@mail.xjtu.edu.cn; xpzhao@msu.edu;

Table of Contents

Supplementary Figures 1-9

Supplementary Notes 1-3

Supplementary Tables 1-2

Supplementary Equations 1-16

Supplementary Note 1: Structure characterization

Supplementary Figure 1 illustrates the structure characterization of the superhydrophilic copper inverse opal wick. For comparison, we also characterized a conventional commercial sintered copper powder sample. As shown in Supplementary Fig. 1(a), the sintered copper powder sample is highly disordered. Supplementary Fig. 1(b) presents a statistical analysis of the small pore diameter distribution, revealing an average diameter of ~ 1.6 μm. Supplementary Fig. 1(c, d) shows the elemental mapping of the evaporator wick, including the distribution of oxygen and copper elements, which indicates a uniform distribution of copper oxide generated during the chemical oxidation process.

We conducted a suction process test of water droplets on the evaporator wick surface at 90° and 180°, as illustrated in Supplementary Fig. 2. A microinjector was employed to precisely control the volume of water droplets. The evaporator wick was positioned on a platform capable of precise height and inclination adjustments. A high-speed camera (Revealer M230) captured the suction processes of the water droplets on the sample surface. In each test, a droplet of predetermined volume (~2 μL) was first generated, after which the platform was gradually raised until the droplet contacted the surface and was subsequently drawn into the wick by capillary pressure. Concurrently, the high-speed camera recorded the suction processes.

The wettability of the micro/nano-structured copper surface was characterized using a KRUSS DSA 100S goniometer via the sessile drop method. A droplet (~4.6 μL) was dispensed using a microsyringe, and the stage holding the sample was gradually raised until the droplet contacted the surface. Upon lowering the stage, the droplet detached from the surface, indicating that the combined adhesion between the droplet and the surface, together with gravitational force acting on the droplet, was lower than the adhesion between the droplet and the needle. To determine the contact angle, the stage was further raised to increase the droplet-surface contact area, thereby enhancing the adhesion force, until the droplet could detach from the needle after lowering the stage, yielding a contact angle of 165.2°. The sliding angle was measured by inclining the stage at a rate of 1°/s, and the droplet slid off rapidly after approximately 8-9 s when the stage's inclination angle (IA) reached ~ 9°, corresponding to a sliding angle of ~ 9°.

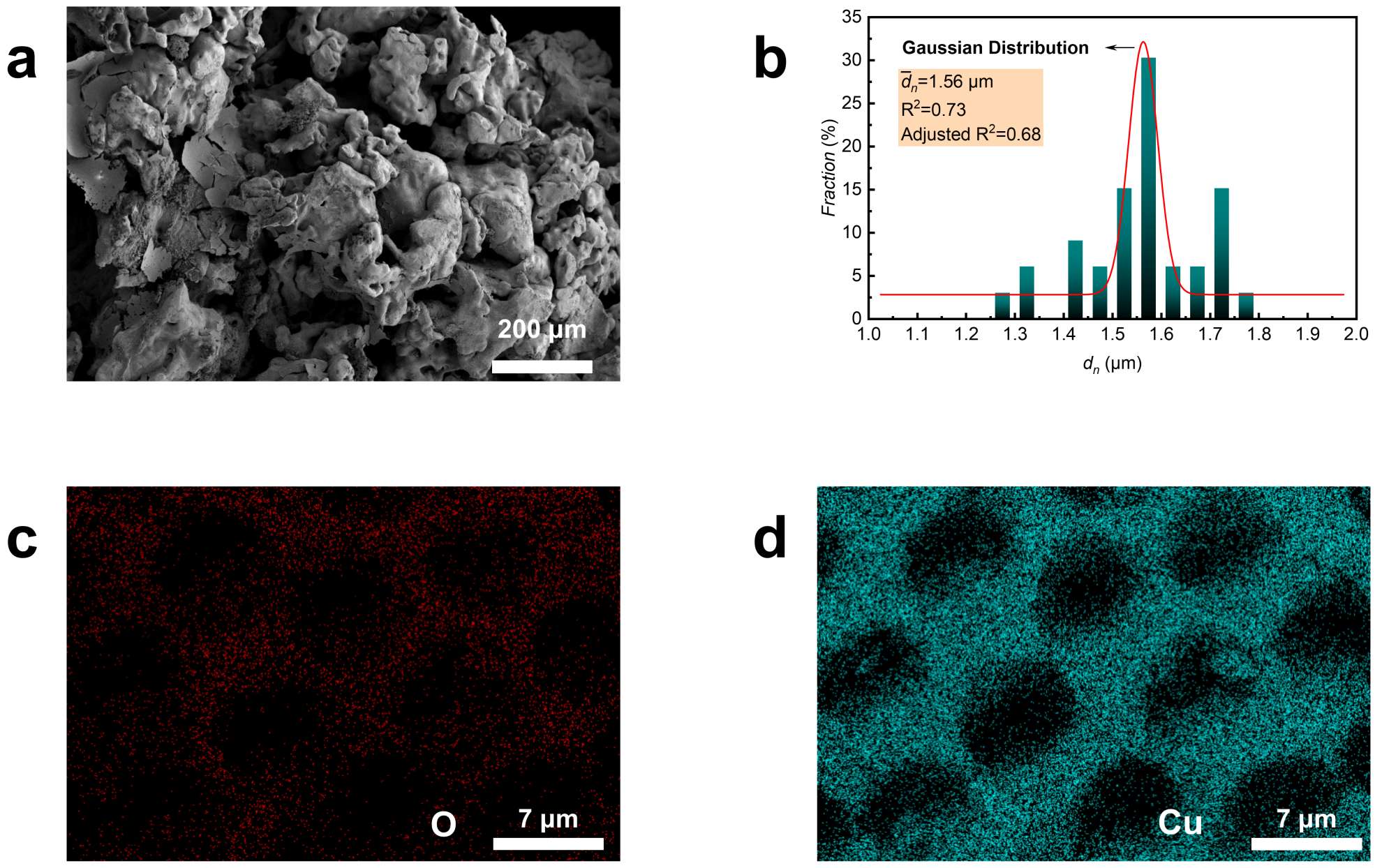

Supplementary Figure 1. Structure characterization of the evaporator wick. **(a)** The structure of a conventional sintered disordered copper powder sample (commercial). **(b)** Statistical analysis of small pore diameter distribution. **(c, d)** Elemental mapping of the evaporator wick reveals the distribution of oxygen and copper elements, indicating a uniform distribution of copper oxide generated during the chemical oxidation treatment.

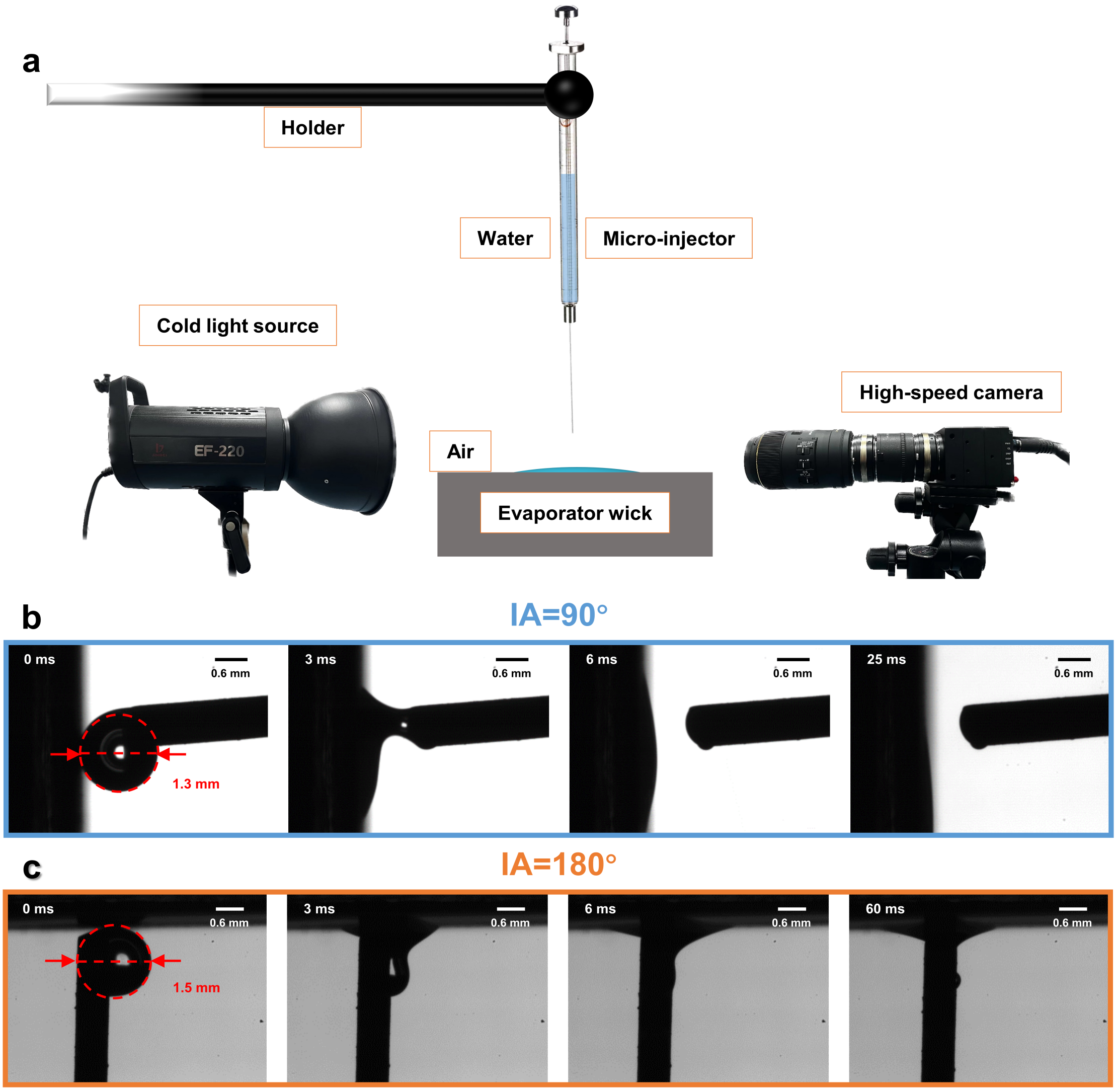

Supplementary Figure 2. Suction process tests of water droplets on the superhydrophilic evaporator wick. **(a)** Schematic of the home-made test rig, which comprises a platform, a high-speed camera (Revealer M230), a cold light, and a microinjector. During the test, the platform was inclined, and a droplet of predetermined volume (~2 μL) was dispensed using the microinjector. The platform was then gradually raised until the droplet contacted the surface, while the high-speed camera recorded the suction processes. **(b, c)** Time-lapse images showing the liquid suction process on the oxidized superhydrophilic copper inverse opal at **(b)** 90°, and **(c)** 180° orientations.

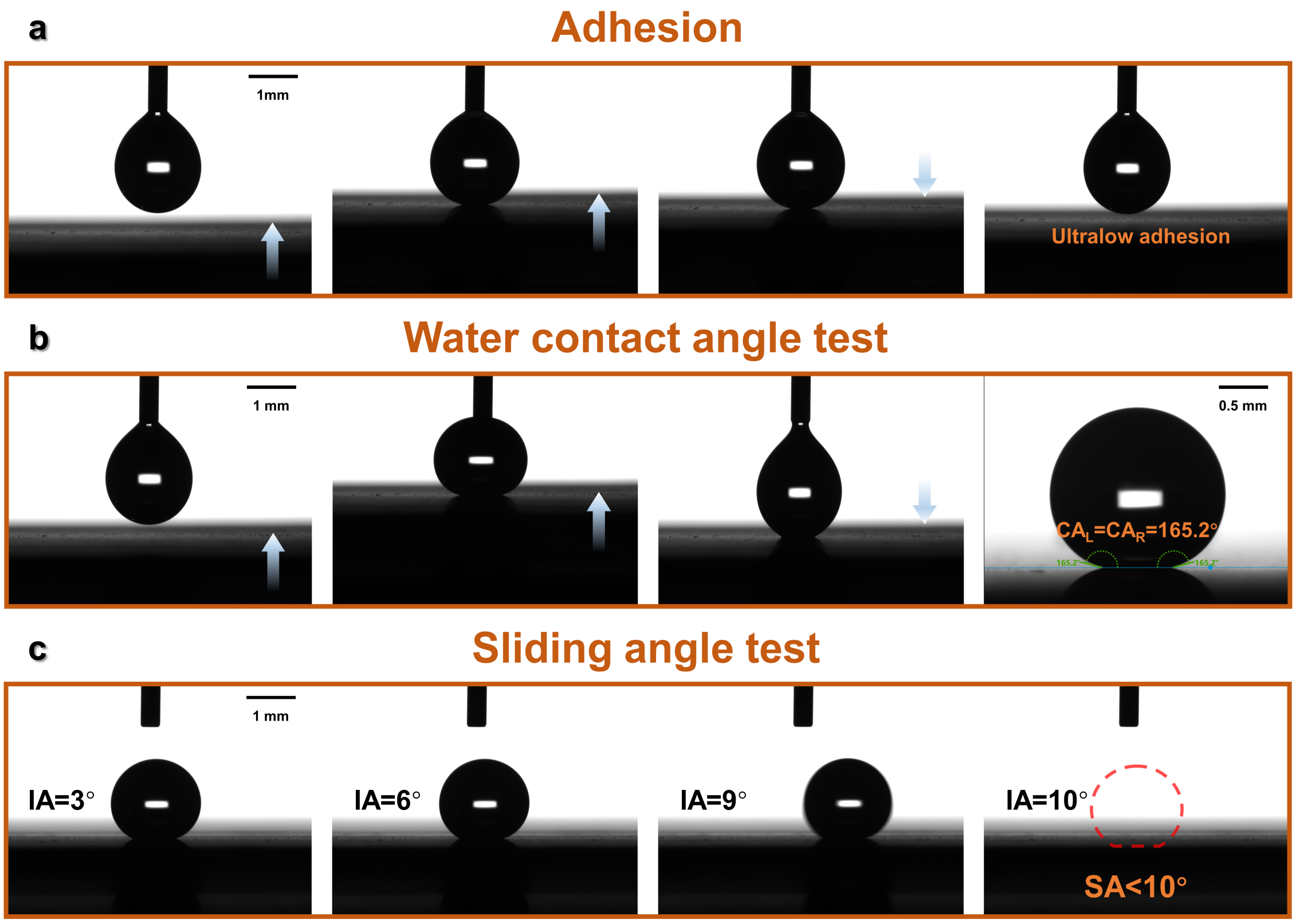

Supplementary Figure 3. Superhydrophobicity characterization of the micro/nano-structured condenser surface using sessile drop method, evaluating **(a)** adhesion, **(b)** static contact angle, and **(c)** sliding angle. **(a)** The droplet detached from the micro/nano-structured surface, indicating ultralow adhesion between the droplet and the surface. **(b)** By pressing the droplet and increasing its contact area with the surface, the droplet was eventually deposited on the surface with a contact angle of 165.02°. **(c)** The platform was inclined at a rate of 1 °/s until the droplet slid away, yielding a sliding angle of ~ 9° in our tests.

Supplementary Note 2: Fabrication of the vapor chamber

The vapor chamber was fabricated by sealing a copper evaporator, a condenser, and a positioning frame (Supplementary Fig. 4(a)). To ensure gas tightness and precise control of the working fluid filling ratio, a custom-built evacuation-filling system was developed. This system comprised a vacuum pump, an injector, a vacuum gauge, and a high-pressure nitrogen cylinder, as shown in Supplementary Fig. 4(b). Seal integrity was validated through a bubble-leak test. The vapor chamber was pressurized to 3 atm with valves V1, V5, and V3 closed, then immersed in a soap solution. No bubble formation was observed during repeated trials, confirming robust sealing performance. The working fluid filling process followed a three-stage procedure: (i) evacuating the system to ~ 50 Pa (V6, V2, V3, and V5 open; V1 and V4 closed), (ii) injecting degassed water via the injector (V1 open; V2, and V6 closed), and (iii) performing a secondary 10-minute evacuation to eliminate residual air before final sealing. The assembled vapor chamber was subsequently subjected to thermal performance testing.

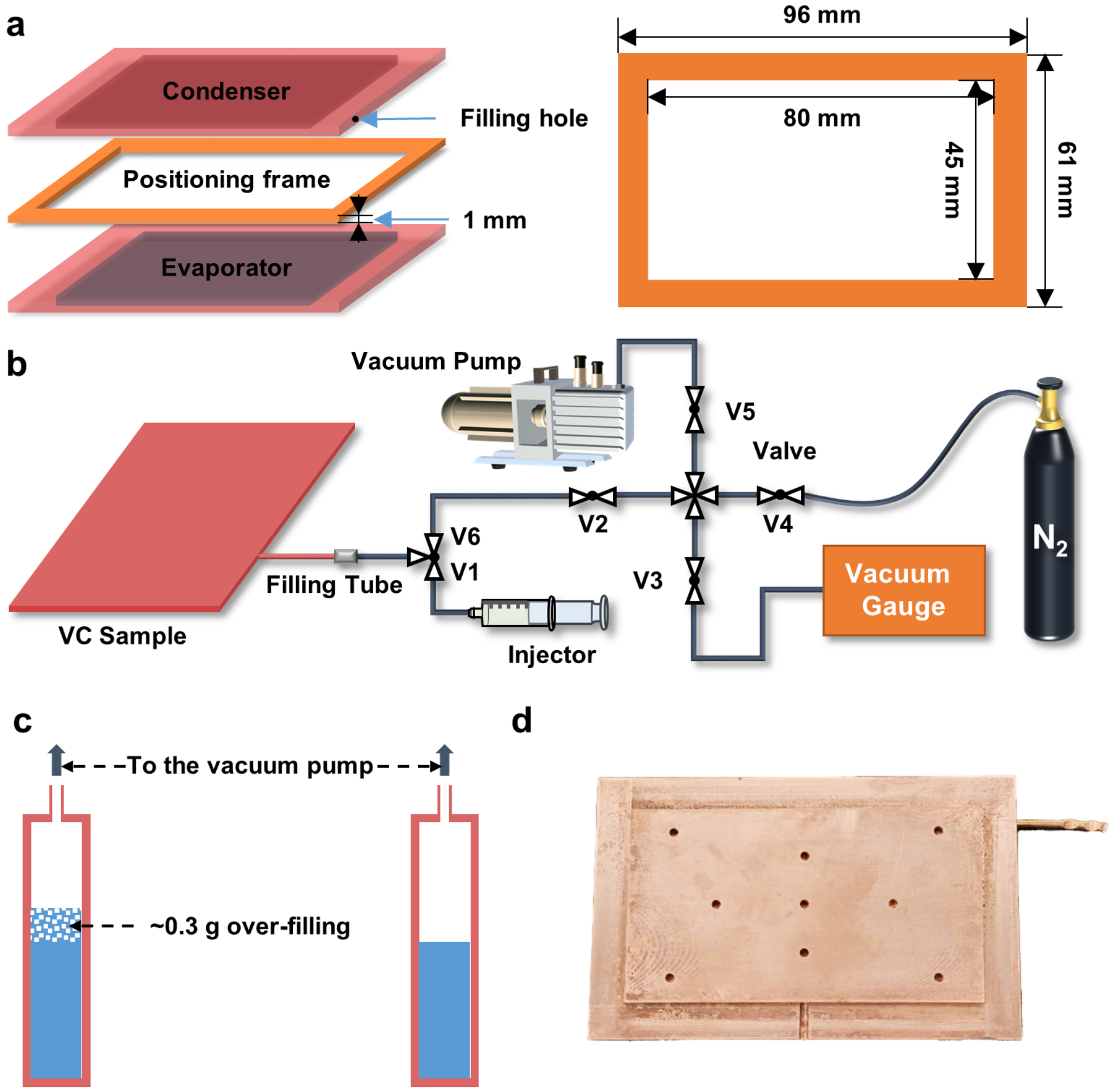

Supplementary Figure 4. Vapor chamber fabrication system. **(a)** Exploded view and dimensions of the components. **(b)** Diagram of the vacuum-assisted liquid filling setup. **(c)** Illustration of the overfilling strategy, where ~ 0.3 g water is initially over injected before the second evacuation. **(d)** Photo of the assembled vapor chamber before testing.

Supplementary Note 3: Thermal dissipation performance test of the vapor chamber

Supplementary Figure 5 shows the custom-designed system, which was used to evaluate the thermal dissipation performance of the vapor chamber, comprising a heating/cooling system, thermal insulation system, and data acquisition system. A Joule-heated cylindrical bar with a diameter of 10 mm was used on the evaporator side to mimic the local high heat flux of high-power electronic devices. A custom-designed water-cooled flat plate was applied on the condenser side to remove the heat. To enhance the cooling efficiency, several fins were machined in the channels of the water-cooled flat plate, as shown in Supplementary Fig. 5(b). A multi-functional indexing instrument was used to control the inclination of the vapor chamber during the tests. Three typical inclinations were tested, including 0°, 90°, and 180°. At 0°, the evaporator was positioned below the condenser; while at 180°, the evaporator was placed above the condenser. At 90°, they were arranged side by side horizontally.

Twenty K-type thermocouples were employed for temperature measurements and data processing. Two of them were embedded in the heating rod with a distance of 5 mm. The heat flux, q, was calculated using Fourier's law, from which the heat transfer rate, Q, was then obtained:

$$q = -\lambda \frac{T_1 - T_2}{l} \tag{1}$$

$$\mathrm{Q} = q \cdot A = -\lambda A \frac{T_1 - T_2}{l} \tag{2}$$

Eighteen thermocouples were uniformly distributed on the evaporator (hot surface) and condenser (cool surface) to evaluate the temperature distribution, as shown in Supplementary Fig. 6. The overall heat dissipation performance of the vapor chamber was characterized by R_{VC}; R_{TU} and ΔT_{Cmax} were used to characterize the temperature uniformity of the condenser surface. The calculations are as follows:

$$T_{\mathrm{Eavg}} = \sum_{i=1}^{9} T_{\mathrm{Ei}} \Big/ 9 \tag{3}$$

$$T_{\mathrm{Cavg}} = \sum_{i=1}^{9} T_{\mathrm{Ci}} \Big/ 9 \tag{4}$$

$$T_{\mathrm{Emax}} = \mathrm{Max}\{T_{\mathrm{E1}}, T_{\mathrm{E2}}, \dots T_{\mathrm{E9}}\} \tag{5}$$

$$T_{\mathrm{Cmax}} = \mathrm{Max}\{T_{\mathrm{C1}}, T_{\mathrm{C2}}, \dots T_{\mathrm{C9}}\} \tag{6}$$

$$T_{\mathrm{Cmin}} = \mathrm{Min}\{T_{\mathrm{C1}}, T_{\mathrm{C2}}, \dots T_{\mathrm{C9}}\} \tag{7}$$

$$R_{\mathrm{VC}} = \frac{T_{\mathrm{Emax}} - T_{\mathrm{Cavg}}}{Q} \tag{8}$$

$$\Delta T_{\mathrm{Cmax}} = T_{\mathrm{Cmax}} - T_{\mathrm{Cmin}} \tag{9}$$

$$R_{\mathrm{TU}} = \frac{\Delta T_{\mathrm{Cmax}}}{Q} \tag{10}$$

The primary source of uncertainty in the experiments arises from the heating system and the temperature acquisition system. The uncertainty is calculated as follows:

$$\Delta \mathrm{Q} = \sqrt{\sum_{i=1}^{2} \left(\frac{\partial Q}{\partial T_{\mathrm{i}}}\right)^{2} (\Delta T_{1})^{2}} \tag{11}$$

$$\Delta\left(T_{\mathrm{Eavg}}\right) = \sqrt{\sum_{i=1}^{9} \left(\frac{\partial\left(T_{\mathrm{Eavg}}\right)}{\partial\left(T_{\mathrm{Hi}}\right)}\right)^{2}} \tag{12}$$

$$\Delta\left(T_{\mathrm{Cavg}}\right) = \sqrt{\sum_{i=1}^{9} \left(\frac{\partial\left(T_{\mathrm{Cavg}}\right)}{\partial\left(T_{\mathrm{Ci}}\right)}\right)^{2}} \tag{13}$$

$$\frac{\Delta(R_{\mathrm{VC}})}{R_{\mathrm{VC}}} = \sqrt{\left(\frac{\Delta(T_{\mathrm{Emax}})}{T_{\mathrm{Emax}} - T_{\mathrm{Cavg}}}\right)^{2} + \left(\frac{\Delta\left(T_{\mathrm{Cavg}}\right)}{T_{\mathrm{Emax}} - T_{\mathrm{Cavg}}}\right)^{2} + \left(\frac{\Delta Q}{Q}\right)^{2}} \tag{14}$$

$$\Delta(\Delta T_{\mathrm{Cmax}}) = \sqrt{\left(\frac{\partial(\Delta T_{\mathrm{Cmax}})}{\partial(T_{\mathrm{Cmax}})}\right)^{2} \left(\Delta(T_{\mathrm{Cmax}})\right)^{2} + \left(\frac{\partial(\Delta T_{\mathrm{Cmax}})}{\partial(T_{\mathrm{Cmin}})}\right)^{2} \left(\Delta(T_{\mathrm{Cmin}})\right)^{2}} \tag{15}$$

$$\frac{\Delta(R_{\mathrm{TU}})}{R_{\mathrm{TU}}} = \sqrt{\left(\frac{\Delta(\Delta T_{\mathrm{Cmax}})}{\Delta T_{\mathrm{Cmax}}}\right)^{2} + \left(\frac{\Delta Q}{Q}\right)^{2}} \tag{16}$$

The uncertainty in these experiments is listed in Supplementary Table 1.

Supplementary Table 1. Uncertainty analysis of the thermal dissipation performance.

Name	Uncertainty
ΔT_{Cmax}, ΔT_{Cmin}, ΔT_{Emax}, ΔT_{Emin}	0.15°C
ΔT_{Cavg}, ΔT_{avg}	0.05°C
$\Delta Q/Q$	7.29%
$\Delta(R_{\mathrm{VC}})/R_{\mathrm{VC}}$	7.57%
$\Delta(\Delta T_{\mathrm{Cmax}})$	0.21°C
$\Delta(R_{\mathrm{TU}})/R_{\mathrm{TU}}$	23.06%

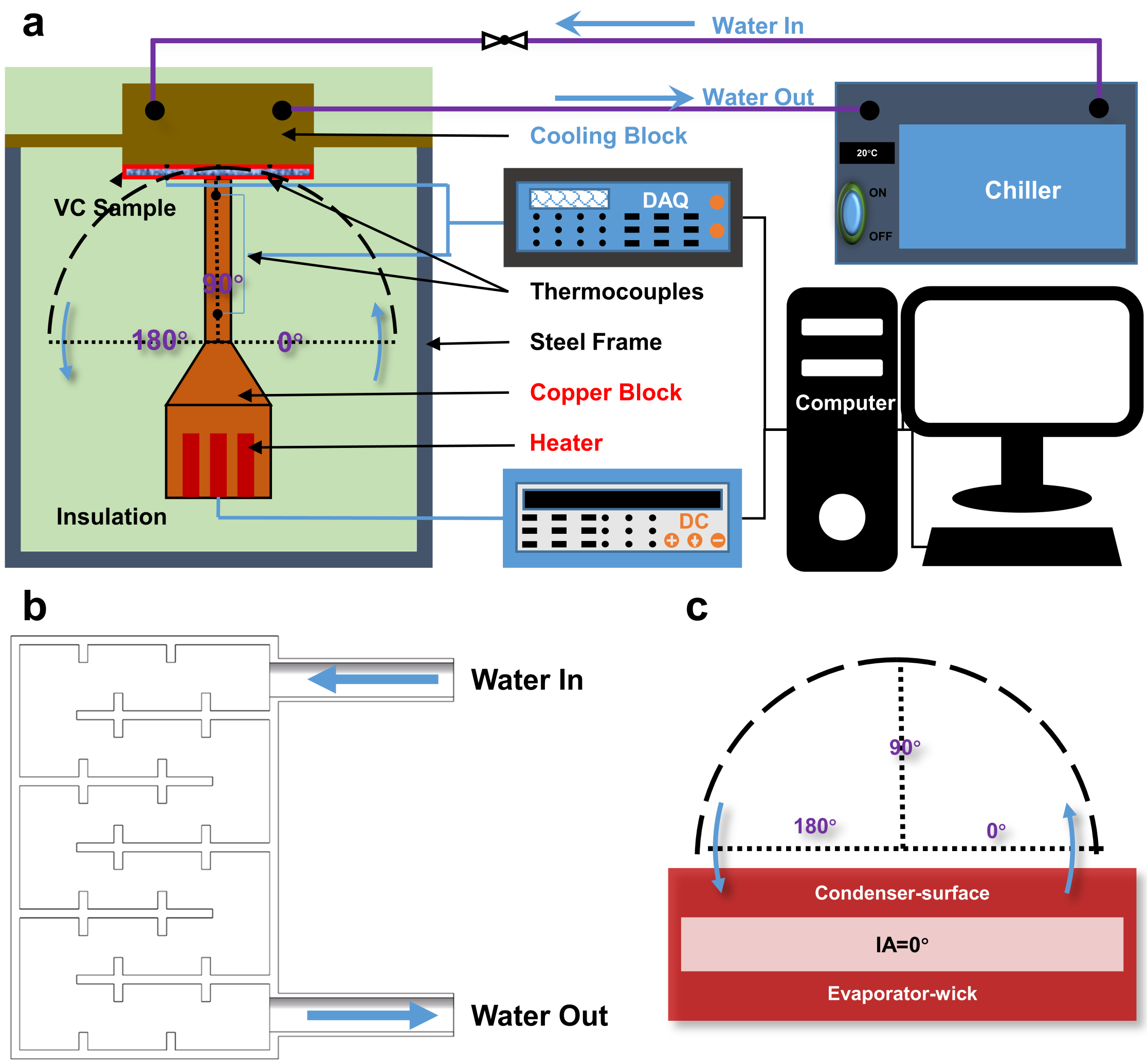

Supplementary Figure 5. Thermal dissipation performance test of the vapor chamber. **(a)** A custom-designed system was used to evaluate the thermal dissipation performance of the vapor chamber, comprising a heating system with a 10 mm diameter to mimic the local high heat flux of high-power electronic devices, and a water-cooled flat plate was used to remove the heat. **(b)** Several fins were machined in the water flow channels of the water-cooled flat plate to enhance the cooling heat transfer. **(c)** A multi-functional indexing instrument was used to control the inclination of the vapor chamber during the tests, three typical inclinations were selected: 0°, 90°, and 180°.

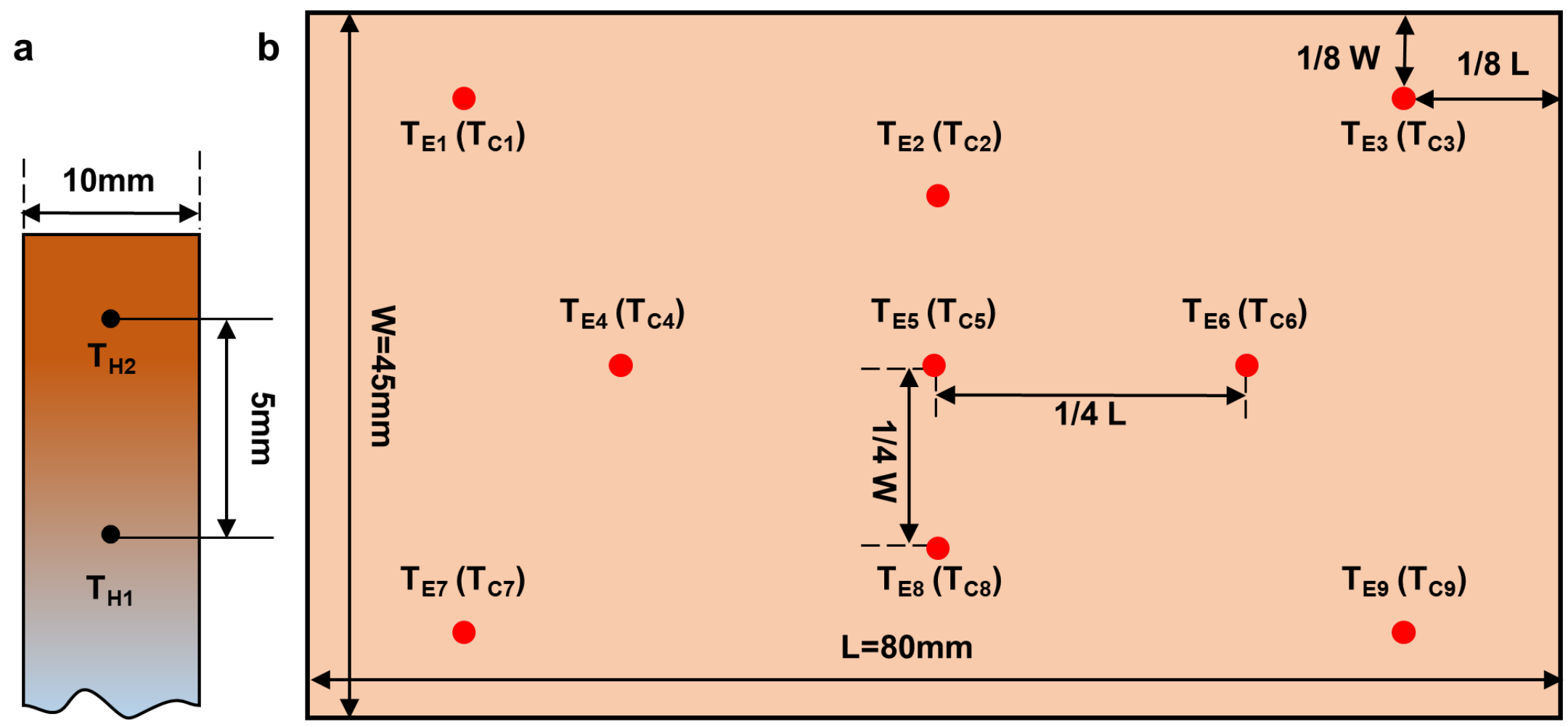

Supplementary Figure 6. The distribution of thermocouples. **(a)** Two thermocouples were embedded in the heating rod, whose values were employed to calculate the heat flux and heat transfer rate from the heater to the vapor chamber. **(b)** Eighteen calibrated K-type thermocouples were uniformly distributed on the evaporator (hot surface) and condenser (cool surface), where T_E denotes the value of the evaporator, with T_C denoting the value of the condenser.

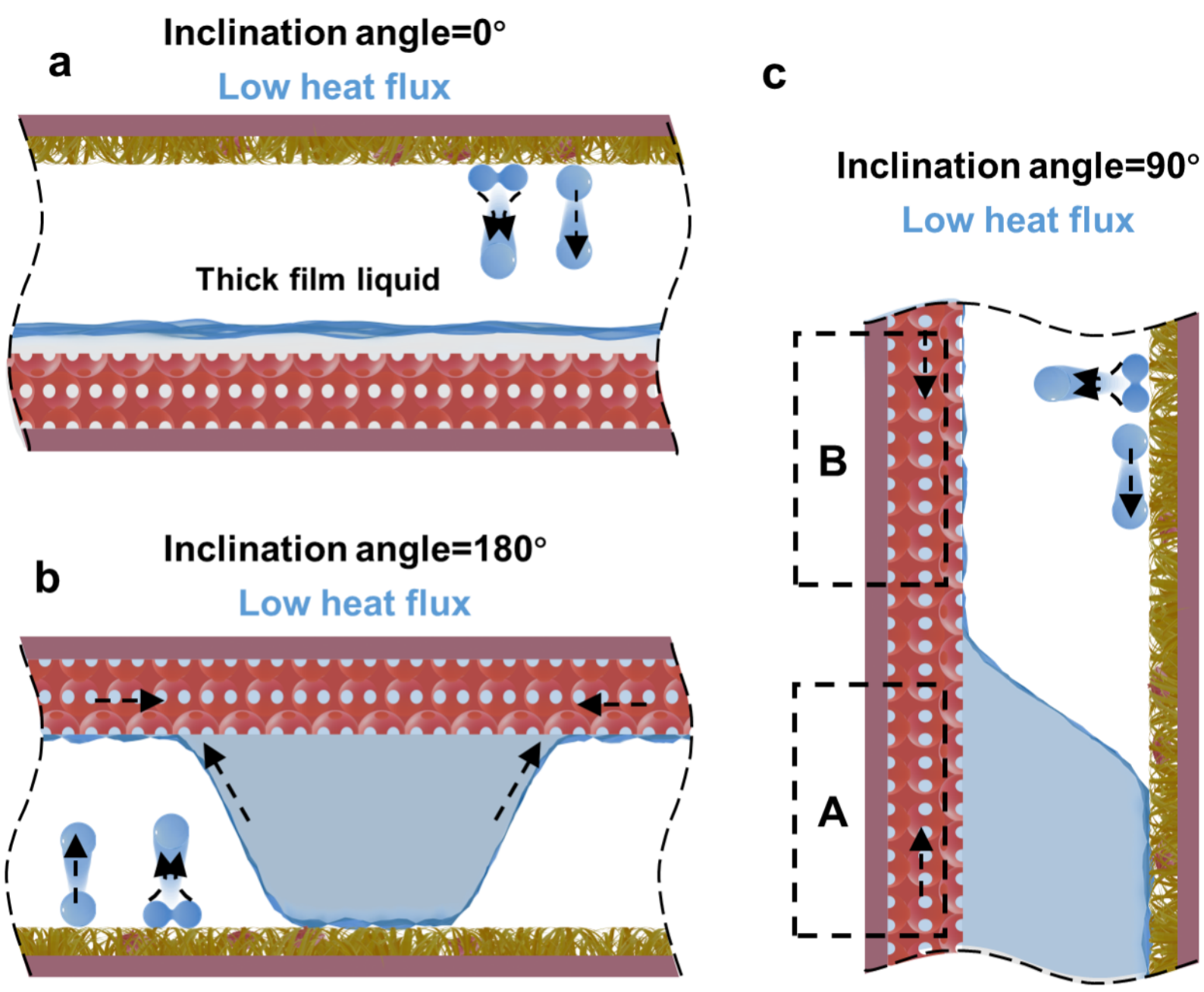

Supplementary Figure 7. Schematic illustrations of phase distribution and liquid-vapor phase change behavior at low heat flux (e.g., $q < 100$ W/cm^2) at **(a)** 0°, **(b)** 180°, and **(c)** 90°, respectively. When at low heat flux, evaporation dominates the phase-change phenomenon.

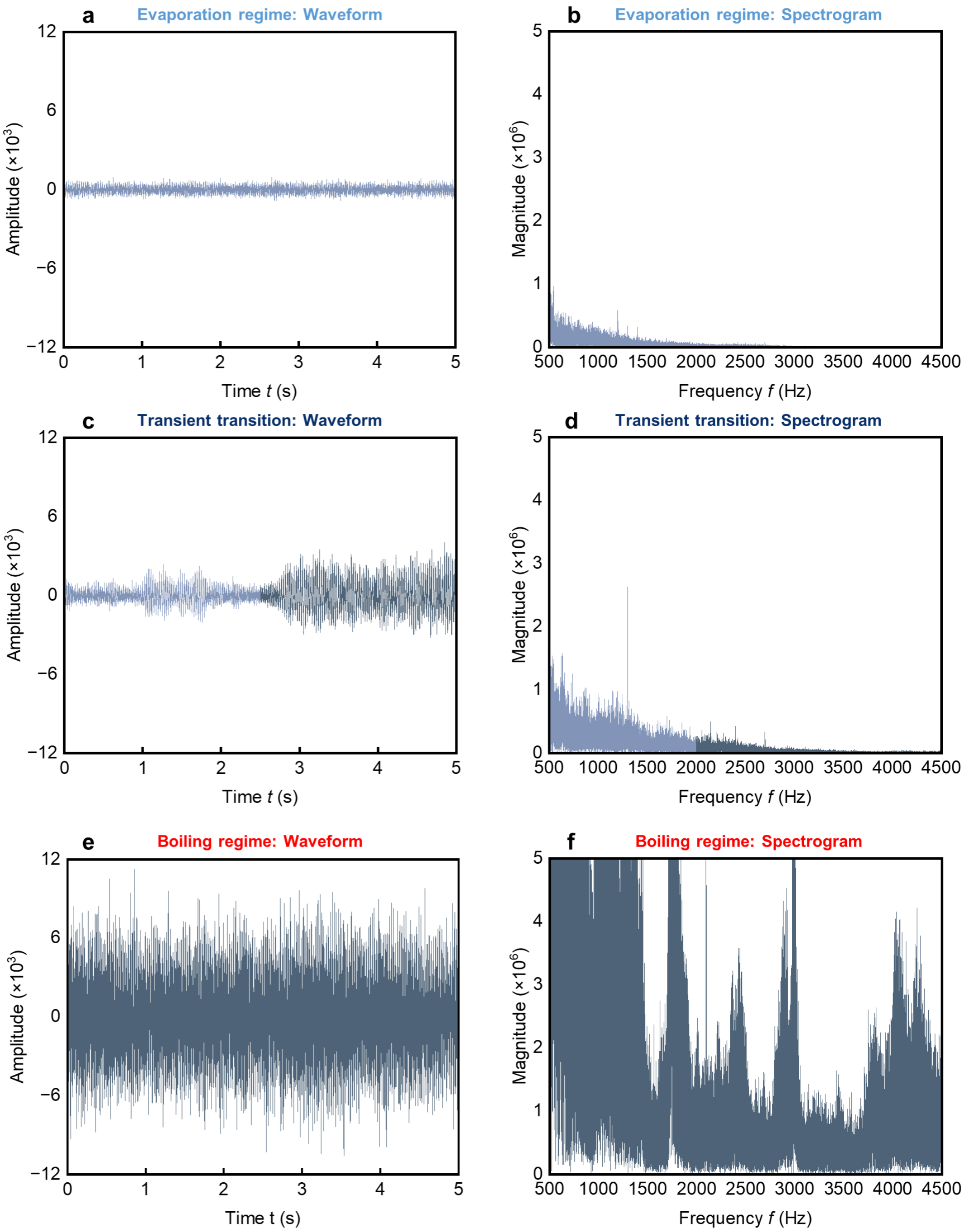

Supplementary Figure 8. Auditory signal analysis during different phase-change regimes. **(a, b)** Waveform and spectrogram of the evaporation regime at ~40 W/cm^2 (180° inclination), showing a flat, quiet signal with minimal high-frequency components. **(c, d)** During the evaporation-to-boiling transition, the waveform becomes more dynamic and the spectrogram exhibits emerging high-frequency peaks. **(e, f)** In the nucleate boiling regime at ~570 W/cm^2 (180° inclination), the waveform shows strong fluctuations and spectrogram displays prominent broadband peaks, indicating vigorous bubble activity.

Supplementary Table 2. Comparison of the thermal dissipation performance of vapor chambers with different designs.

Refs	Evaporator structure	Condenser structure	Filling condition	Heating/Cooling area (cm^2)	Cooling condition	*Q* (W)	*q* (W/cm^2)
Present	Superhydrophilic copper inverse opal	Superhydrophobic micro/nano-structured copper surface	50%	0.79/36	20 °C, 13.4 L/min	**471**	**600**
2	Mastoid process array sintered copper particle	Copper surface with nanosheets	30%	1/84	40 °C, 9.1 L/min	140	100
14	Sintered powder with radial grooves	Sintered powder layer	40%	1/144	30 °C, 100 L/min	280	280
24	Superhydrophilic copper mesh	Superhydrophilic copper mesh	51%	0.1/50	1.4 L/min	49	490
25	Biomimetic copper forest	Biomimetic copper forest	71%	1/36	Natural air convection	6	6
32	Wettability patterned surface	Wettability patterned surface	20%	0.9/25	19 °C, 1.34 L/min	199	220
33	Porous copper foam	Porous copper foam	15%	0.79/79	Forced air convection	275	350
34	Copper foam	Copper foam	30%	0.79/79	Forced air convection	170	216
35	Sintered copper particles	Sintered copper particles	35%	1.1/81	15 L/min	50	44
36	Copper mesh	Copper mesh	Not given	0.16/81	Forced air convection	30	188
37	Sintered copper powders	Layered copper screens	Not given	1/84	40 °C, 9.1 L/min	387	387
38	Copper mesh	Copper mesh	3.4 mL	4.41/90	Forced air convection	360	80
39	Sintered powder layers	Sintered powder layers	Not given	1/81	26 °C, 0.1 L/min	300	300

Refs	Evaporator structure	Condenser structure	Filling condition	Heating/Cooling area (cm^2)	Cooling condition	Q (W)	q (W/cm^2)
40	Biporous sintered powder	Superhydrophobic copper	1.73 g	2.3/49	35 °C	141	220
41	Micro-grooved wick with reentrant cavity array	Micro-grooved wick with reentrant cavity array	40%	7.1/900	20 °C, 1 L/min	150	21
42	Hybrid wicks	Hybrid wicks	50%	1/84	Maintained 40 °C	350	350
43	Sintered wick structure	Sintered wick structure	45%	1.2/50	35 °C, 4 L/min	120	100
44	Leaf-vein-like fractal network	Leaf-vein-like fractal network	63%	9.6/63	Forced air convection	90	9
45	Sintered multi-artery particle	Copper mesh	~20 mL	1/84	~30 °C, 15 L/min	262	262
46	Porous wick with dimple structure	Porous wick with dimple structure	60%	0.25/25	Forced air convection	50	200
47	Groove wick	Groove wick	1.6 g	3.1/57	Forced air convection	118	37
48	Superhydrophilic sintered copper powder	Wettability-patterned surface	22%	0.9/26	21 °C	155	172
49	Homogeneous copper foam	Fractal network microchannels	63%	4/38	60 °C, 1.7 L/min	140	35
50	Sintered copper powder	Sintered copper powder	45%	16/96	20 °C	200	13
51	Micropillar	Superhydrophobic copper	3.4 mL	0.1/Not given	Maintained 50 °C	51	400
52	Sintered copper screen mesh	Copper surface	75%	16/36	20 °C, 0.15 L/min	161	10

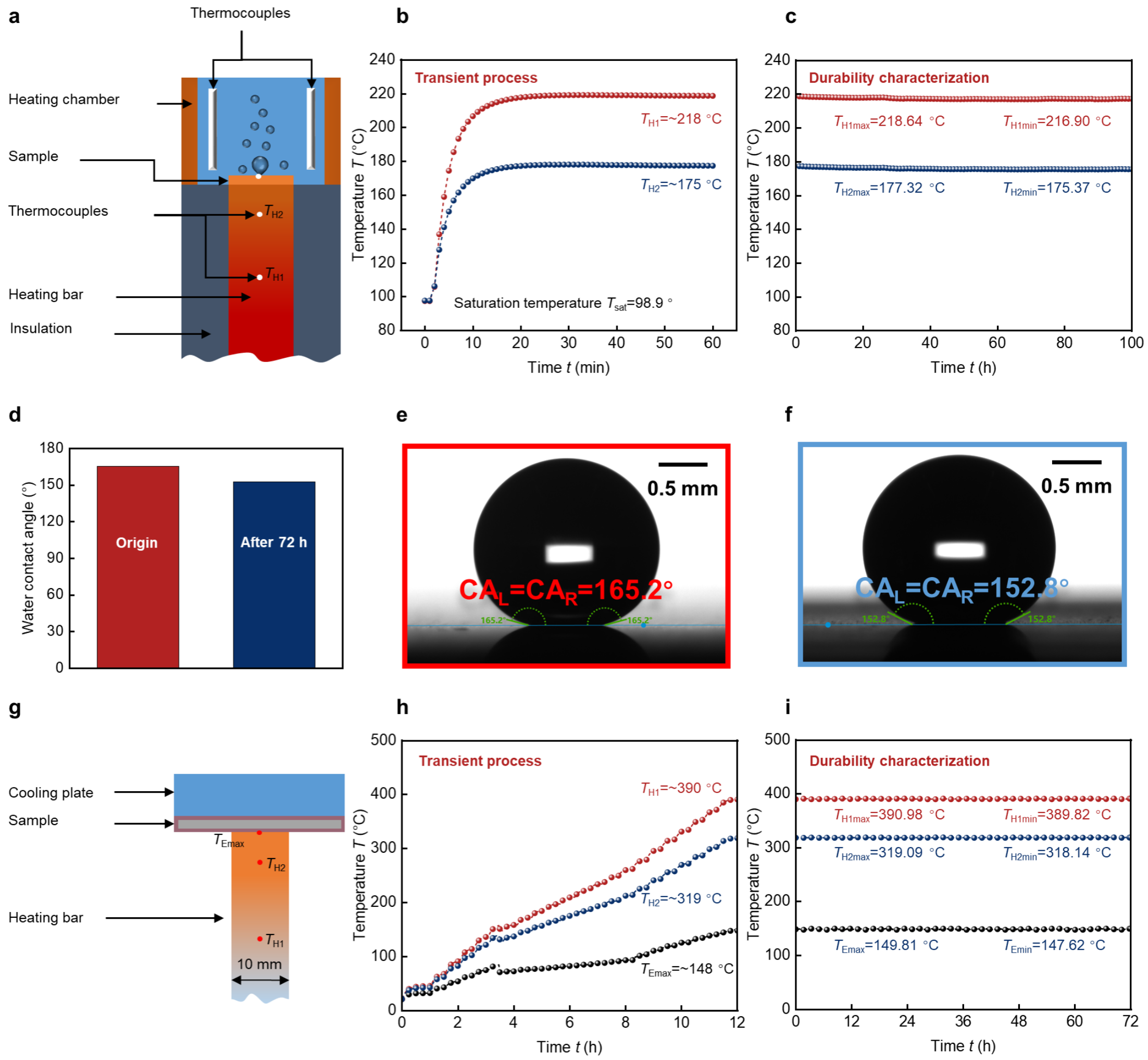

Supplementary Fig. 9. Durability assessment of the structure and vapor chamber. **(a)** Schematic of the pool boiling durability test setup, featuring a liquid chamber, heating module, thermocouples, and insulation. **(b)** Transient temperature responses of the heating bar at a heat flux of ~172 W/cm^2. **(c)** Long-term stability of the heating bar temperature, showing minimal degradation after 100 hours of continuous boiling, confirming the structural robustness of the superhydrophilic biporous wick. **(d)** Contact angle comparison of the superhydrophobic condenser surface before and after >72 h water immersion, showing minimal degradation. **(e, f)** Representative contact angle measurements: **(e)** Original contact angle of ~165.2°, and **(f)** still retained >150° after 72 h immersion, confirming durable superhydrophobicity. **(g)** Schematic of the vapor chamber test configuration. **(h)** Transient temperature evolution at the heating bar and evaporator center with increasing applied heat flux. **(i)** Durability of the vapor chamber over 72 hours operation, showing stable performance with overall thermal resistance maintained at ~0.22 °C/W.
